# A novel nondestructive diagnostic method for mega-electron-volt ultrafast electron diffraction

**DOI:** 10.1038/s41598-019-53824-9

**Published:** 2019-11-20

**Authors:** Xi Yang, Junjie Li, Mikhail Fedurin, Victor Smaluk, Lihua Yu, Lijun Wu, Weishi Wan, Yimei Zhu, Timur Shaftan

**Affiliations:** 1 Brookhaven National Laboratory, Upton, NY 11973 USA; 2 ShanghaiTech University, Shanghai, China

**Keywords:** Characterization and analytical techniques, Characterization and analytical techniques

## Abstract

A real-time, nondestructive, Bragg-diffracted electron beam energy, energy-spread and spatial-pointing jitter monitor is experimentally verified by encoding the electron beam energy and spatial-pointing jitter information into the mega-electron-volt ultrafast electron diffraction pattern. The shot-to-shot fluctuation of the diffraction pattern is then decomposed to two basic modes, i.e., the distance between the Bragg peaks as well as its variation (radial mode) and the overall lateral shift of the whole pattern (drift mode). Since these two modes are completely decoupled, the Bragg-diffraction method can simultaneously measure the shot-to-shot energy fluctuation from the radial mode with 2·10^−4^ precision and spatial-pointing jitter from the drift mode having wide measurement span covering energy jitter range from 10^−4^ to 10^−1^. The key advantage of this method is that it allows us to extract the electron beam energy spread concurrently with the ongoing experiment and enables online optimization of the electron beam especially for future high charge single-shot ultrafast electron diffraction (UED) and ultrafast electron microscopy (UEM) experiments. Furthermore, real-time energy measurement enables the filtering process to remove off-energy shots, improving the resolution of time-resolved UED. As a result, this method can be applied to the entire UED user community, beyond the traditional electron beam diagnostics of accelerators used by accelerator physicists.

## Introduction

In recent years, there has been a growing interest in developing single-shot mega-electron-volt (MeV) UED systems^1–12^. The main advantages of relativistic electron diffraction as compared to the more common diffraction with electron energies in the 100 keV range are reduced space charge effects and higher penetration depth. Also, UED can resolve much finer structural details compared to X-rays due to the hundreds-fold shorter wavelength of electrons in the required sub-picosecond timescale. However, single-shot imaging at high spatial-resolution with small beam size on the sample presents significant challenges. It requires much brighter electron sources. For instance, it is estimated that the RF gun needs to be three orders of magnitude brighter than the present state-of-the-art to outrun beam-induced damage in biomolecular single-particle imaging, achieving “diffraction-before-destruction”^13^. Multi-shot operation, on the other hand, can significantly reduce the demand for the beam brightness, but with much lower tolerances to shot-to-shot energy and spatial-pointing fluctuations. Furthermore, time-resolved MeV UED has been recently established as the probe for ultrafast laser-induced changes of long-range order for some materials^14^. To characterize the variation of a lattice constant in the order of 10^−3^ after the pump excitation, the electron beam energy has to be measured shot-by-shot with a precision in the order of 10^−4^ concurrently during the experiment. Also, the real-time energy measurement enables the filtering process to remove off-energy shots, improving the resolution of time-resolved UED. Furthermore, multiple single-shot images with the same delay after the pump excitation can be recorded. By partially illuminating the sample with the pump laser, the diffraction pattern includes both Bragg peaks with and without the pump excitation. Since the Bragg peaks without excitation provide the shot-to-shot calibration of the electron beam energy, the diffraction pattern with pump excitation can be normalized by this real time measured beam energy. By doing so, the error caused by the shot-to-shot energy fluctuation can be reduced to the measurement precision. Offline summation of these single-shot images with the energy correction can simultaneously take advantage of high precision of the single-hot and high intensity of the accumulation. This application has great potential to benefit a much broader UED user community, is no longer limited to accelerators and particle beam as the diagnostic method. To meet all these requirements, we need a real-time, nondestructive, electron beam energy and spatial-pointing jitter monitor to characterize the shot-to-shot energy fluctuation and energy spread of the electron beam.

In this article, we report our proof-of-principle experiment - characterizing the shot-to-shot energy and spatial-pointing jitter and energy spread of the electron beam for UED and UEM *via* a novel Bragg-diffraction method (BDM). This diagnostic method utilizes the existing high-charge high-brightness low-energy electron source developed at Brookhaven National Laboratory (BNL) with the capability of generating ~3.3 MeV electron bunches at 10 pC charge (0.62·10^8^ electrons) and 0.1 to 1 ps bunch length^10,11^. The information of the electron beam energy, energy fluctuation and spatial-pointing jitter is intrinsically encoded to the shot-to-shot diffraction image. Without introducing any perturbation to the ongoing experiment, we were able to simultaneously measure the shot-to-shot energy fluctuation and spatial-pointing jitter of the electron beam in real-time via eigen-decomposing the variation of the diffraction pattern to two decoupled modes (radial and drift). Also, we obtained the dispersion of the beamline optics at the detector via analyzing the correlation between the shot-to-shot spatial-pointing jitter and energy fluctuation. Beyond tracking changes of the intensity, position, and width of diffraction patterns^15^, we applied the dispersion and Bragg peak width to extract the electron beam energy spread. The preliminary result of the measured electron beam energy spread agrees reasonably well with Impact-T simulation^16^ and with the direct beam-size measurement without crystal diffraction. The nondestructive measurement of the electron beam parameter and beamline optics opens the possibility of online minimization of the shot-to-shot energy and spatial-pointing jitter and energy spread of the electron beam in real-time, which are crucial for the future single-shot UED and UEM development. This is impossible with the conventional dipole-based diagnostic tool. We have experimentally demonstrated that the BDM can provide a nearly complete set of beam-based diagnostic information for online optimization of the RF system stability and minimization of the dispersion at the detector. This is especially important for high charge electron beams for future UED and UEM experiments.

## Results

**Figure 1:**
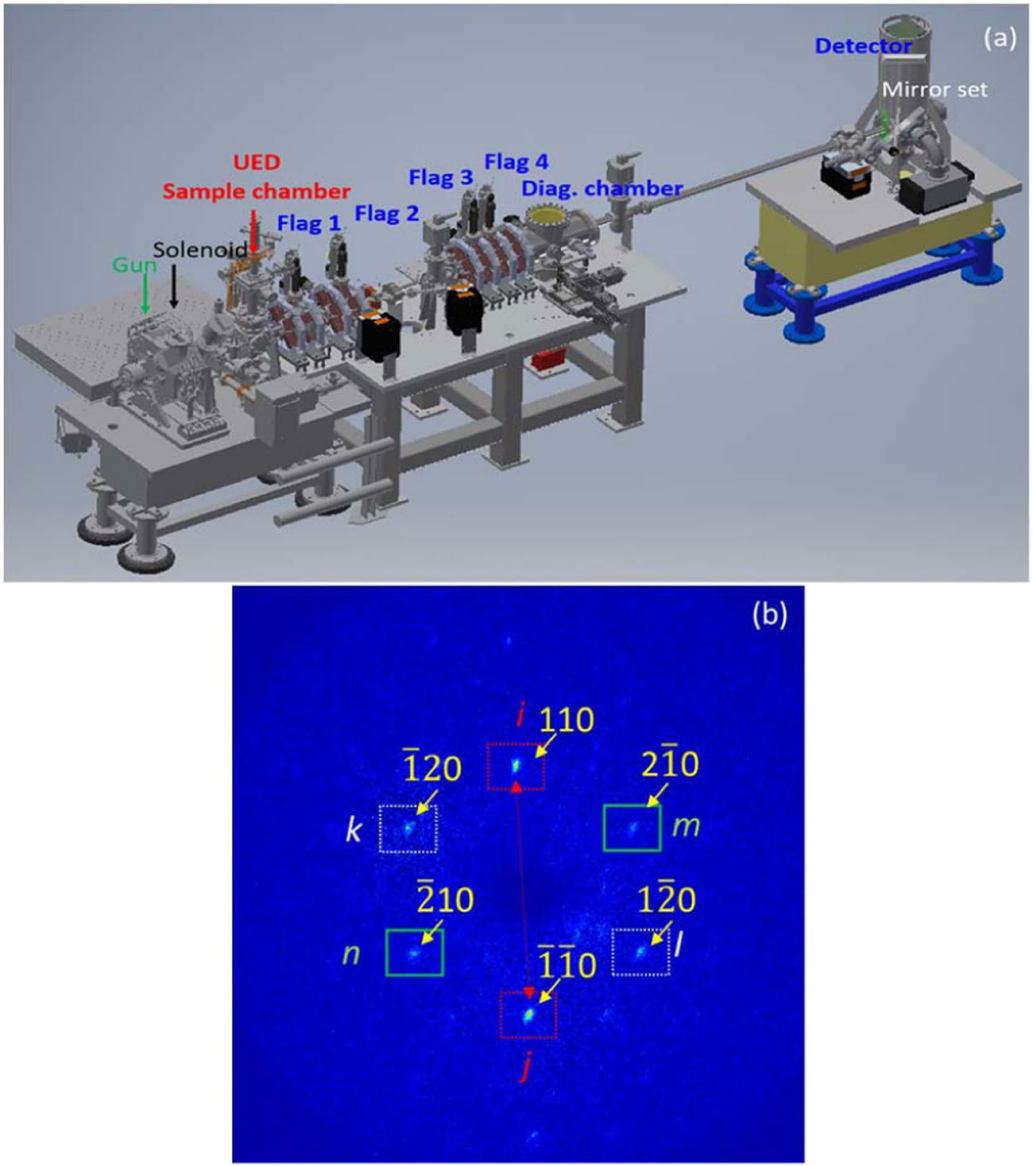
(**a**) Schematic layout of the UED beamline with positions of the UED sample chamber, YAG screens and detector. All the quadrupoles were turned off. (**b**) Single-shot Bragg diffraction image on the detector. Miller indexes of Bragg peaks used in data analysis are labeled (yellow).

The schematic of the UED setup is shown as Fig. 1(a). The detector is a CCD array with 512 pixels in x and 512 pixels in y. The x and y calibrations are the same 60 µm per pixel^2^. The mirror set is part of the detector system, including a phosphor screen (green) and a downstream mirror with a hole in the middle (grey). The hole allows the background noises generated by the core of the non-interacted electron beam and by the dark current to pass through the mirror to the dump. In this setup, the detector resolution is not limited by the phosphor screen thickness which is less than 100 µm; instead, it is limited by the calibrated pixel size of the detector.

The repetition rate of the electron shots is 5 Hz. It experimentally evidence that there might be huge benefits for using pulsed electrons to probe beam-sensitive soft materials^17,18^. Since there is a 200 ms time between two consecutive electron beam exposures, the sample can often recover from beam induced temporary impairment such as bond breaking^19–22^. Besides, the beam size at the UED sample chamber is ≥100 µm^10^, which is significantly larger compared to the traditional Transmission Electron Microscopy (TEM). In the experiment, we haven’t observed any beam-induced degradation of the diffraction sample. But with increase of the repetition rate, radiation induced target damage can be an issue, which requires careful consideration on the choice of target material and its damage mechanism.

The Bragg peaks of a diffraction image are formed through the summation of the intensity distribution of all diffracted electrons (e.g. Fig. 1(b)). The diffraction pattern of a single electron is determined by the constructive interference governed by Bragg’s law 2*d* sin*θ* = *nλ*, where *θ* is the incident angle, *d* is the crystal interplanar distance, *λ* is the deBroglie wavelength, *n* is the order of Bragg reflections. To increase the measured energy resolution, we use single-shot images in the data analysis, which helps to mitigate the reduction of the resolution due to the shot-to-shot variation of the diffraction pattern. The drawback of this single-shot approach is that only a few low-order Bragg peaks are intense enough for reliable data analysis. The accuracy of finding the positions of those peaks determines the ultimate measured energy resolution.

We develop a numerical procedure that can be used to analyze the diffraction pattern by refining each Bragg peak with a Gaussian function. The distances between Bragg peaks are calculated from such refined peak positions. To achieve the highest-possible resolution, we use noise reduction techniques with a dynamically adjustable background and a user-defined region of interest (ROI) and we apply averaging over the same order of Bragg reflections. Before choosing two Bragg peaks (*i* and *j* in Fig. 1(b)) with simultaneously the largest separation, the highest peak intensities and the same reflection order (*n*_*i,j*_ = *n*) as well as crystal interplanar distance (*d*_*i,j*_ = *d*) for the data analysis, we compare the result from this Bragg-peak pair (*ij*) to the result from all three Bragg-peak pairs with the same reflection order (*ij, kl*, *mn*). In most shots, the differences are within the energy resolution 2.10^−4^. Since Bragg peak pairs *kl* and *mn* are occasionally weak in intensities, including them in the calculation doesn’t improve the accuracy and could make it worse. This is the reason why we choose to use the peak pair with the highest intensities. The separation between the peak pair *ij* (*D*_*ij*_) is determined by the interplane distance *d*, the distance between the sample and the detector *L*_*S2D*_ (=3.54 m) and the electron beam energy *E*, shown as Eq. (1).

1 $${D}_{ij}(E,d)={L}_{S2D}\cdot \{\tan \,[2{\theta }_{i}(E,d)]-\,\tan \,[2{\theta }_{j}(E,d)]\}={L}_{S2D}\cdot 2\cdot \,\tan [2si{n}^{-1}(\frac{n\cdot \lambda (E)}{2d})]\approx {L}_{S2D}\cdot \frac{2n\cdot \lambda (E)}{d}$$

This separation can be used to calibrate the shot-to-shot energy jitter based on Eq. (2).

2 $$\frac{\Delta {D}_{ij}}{{D}_{ij}}=\frac{\Delta \lambda }{\lambda }=-\frac{\Delta {\rm E}}{{\rm E}}$$

We use Levenberg-Marquardt method in Matlab to refine the peak position. The standard deviation of peak position can be estimated from the covariance matrix^23,24^, which is less than 0.05 pixel in our case. We examine the distances of Bragg-peak pairs *ij*, *kl* and *mn*, e.g. 220.01, 219.96 and 220.03 pixels respectively. They differ from each other within the measurement precision of the peak positions. Thus, the relative accuracy *∆D*/*D* is about 0.05/220 ≈ 2.3 10^−4^, which is an order of magnitude smaller than the shot-to-shot energy jitter.

**Figure 2:**
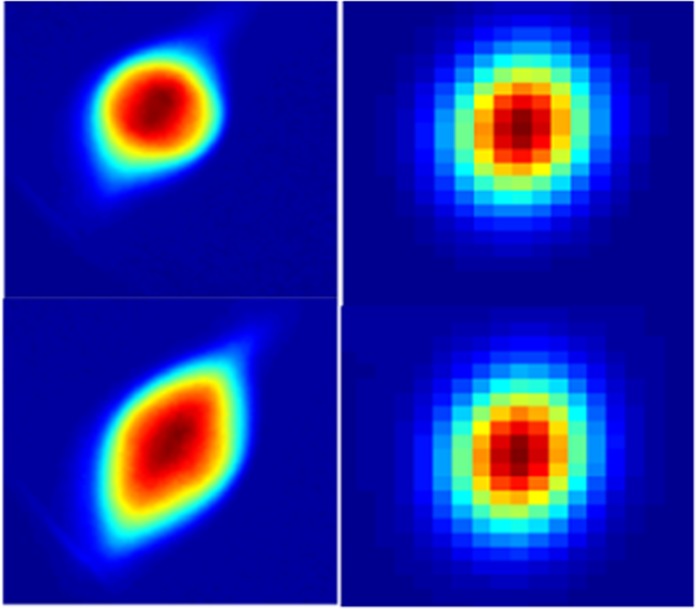
Two different electron beam profiles 1 (top left) and 2 (bottom left) at the detector and their corresponding Bragg peaks with the same Miller index (110): for the beam profile 1 (top right) and for the beam profile 2 (bottom right).

We experimentally compared several different transverse shapes of the incident electron beam, together with their corresponding diffraction patterns. E.g., Fig. 2 shows two different electron beam profiles 1 (top left) and 2 (bottom left) at the detector and their corresponding Bragg peaks with the same Miller index (110): for the beam profile 1 (top right) and for the beam profile 2 (bottom right). The resulting energy resolution does not change among those cases. The reason is that the energy resolution depends on the measurement precision of the center positions of Bragg peaks. Once these peaks are symmetric, the actual shape of the peak does not affect the center position therefore the energy resolution. Once the sample stays the same among different consecutive electron beam exposures which is the case in the experiment, the crystal interplanar distance *d* is constant. Based on Eq. (1), the shot-to-shot energy jitter is the only source causing the variation of ∆*D*/*D*. The uncertainty coming from the fitted error of the center positions of those Bragg peaks determines the ultimate measured energy resolution. There is no need for the detail information of crystal interplanar distance and sample-to-detector distance since we are interested in the shot-to-shot energy jitter instead of the absolute beam energy.

**Figure 3:**
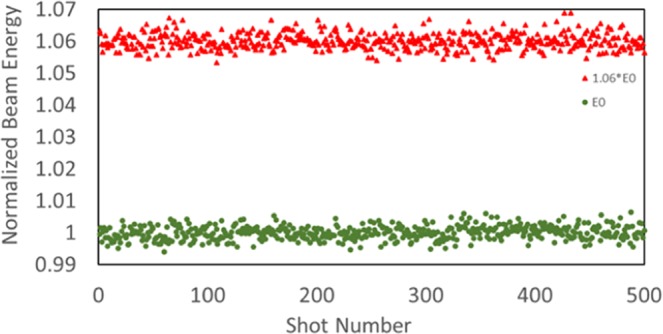
The shot-to-shot energy variation measured at two different electron beam energies: 1.06*E*_0_ with the fluctuation 0.239% in standard deviation (red) and *E*_0_ with the fluctuation 0.216% (green). They are similar. The shot-to-shot beam energy is normalized by the reference energy which is the average value of all 500 shots with the operational RF setting.

There are two basic components associated with the shot-to-shot fluctuation of the diffraction image. We call the mode which corresponds to the expansion and contraction of the diffraction image in the radial direction with respect to the image center the radial mode, and the other mode representing the overall lateral shift of the diffraction image the drift mode. The shot-to-shot energy jitter causes the radial motion of the diffraction image and contributes to the radial mode only, as shown in Fig. 3. Since there are no magnetic or electrical elements between the sample and the detector, aside from orbit correctors, the distance between Bragg peaks is only determined by the electron beam energy, distance between sample and detector and sample properties. The correctors are weak (<1mrad) and fed by highly stable (10^−5^) power supplies, with the perturbation ≤0.1 µrad. The sample used in the measurement is a 2D thin film of TaS_2_ with CDW, which was exfoliated from a bulk material. The film has a thickness range of 30 to 80 nm.

The drift mode includes both the spatial-pointing jitter and the dispersive jitter due to the combinatory effect of the non-zero dispersion at the detector and the shot-to-shot beam energy fluctuation. Non-zero dispersions (*η*_*x,y*_) at the detector are caused by the steering from the earth magnetic field^10,11^, orbit correctors and beam off-center at the solenoid. When the beam energy fluctuates shot-by-shot, the non-zero dispersions at the detector will cause the transverse motion Δ*R* of the diffraction image based on Eq. (3).

3 $$\Delta R({\eta }_{x},{\eta }_{y},\frac{\Delta {\rm E}}{{\rm E}})=\sqrt{\Delta {x}^{2}+\Delta {y}^{2}}=\sqrt{{({\eta }_{x}\cdot \frac{\Delta {\rm E}}{{\rm E}})}^{2}+{({\eta }_{y}\cdot \frac{\Delta {\rm E}}{{\rm E}})}^{2}}=\sqrt{{{\eta }_{x}}^{2}+{{\eta }_{y}}^{2}}\cdot \frac{\Delta {\rm E}}{{\rm E}}$$

**Figure 4:**
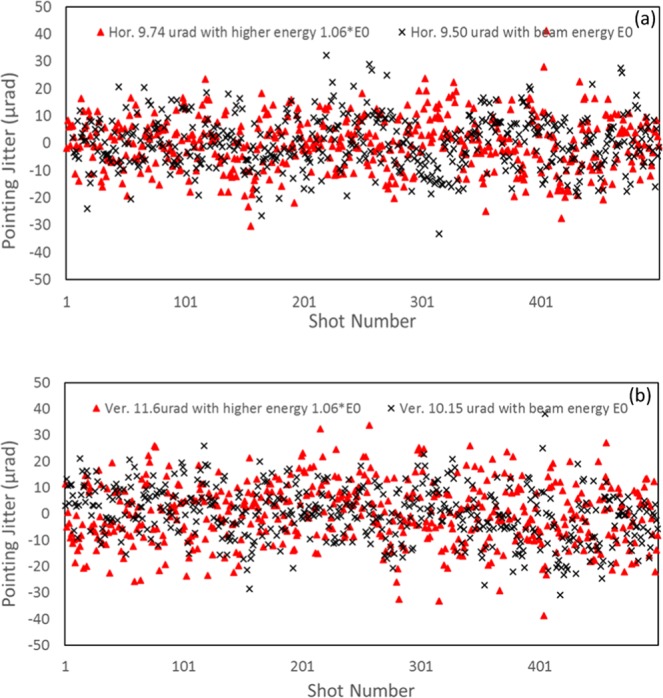
The shot-to-shot pointing jitter measured at two different electron beam energies: 1.06*E*_0_ (red) and *E*_0_ (black) in the case with comparably small energy jitters. The results are similar, about 10 µrad spatial-pointing jitter in both horizontal (**a**) and vertical (**b**) directions.

In case the shot-to-shot energy jitter is small (<0.3%) without any slow drift or periodic oscillation, the drift mode mainly comes from the spatial-pointing jitter of the UED system (e.g. shot-to-shot laser pointing jitter at the cathode), as shown in Fig. 4. However, even when the shot-to-shot energy jitter is large (addressed later in the paper), the spatial-pointing jitter still can be extrapolated from the uncorrelated part of the shot-to-shot ‘pointing jitter *vs* energy jitter’. The results are similar in both cases, about 10 µrad in both horizontal and vertical directions. Here, we focus on the situation without any slow drift and large energy osciallations. The spatial-pointing jitter was measured *via* the shot-to-shot random variation of the drift-mode position *x, y*_*current*_ with respect to the reference position *x, y*_*ref*_ at the detector Δ*x*, *y*_*uncor*_ = *x*, *y*_*current*_ −*x*, *y*_*ref*_. The reference position is obtained by averaging drift-mode positions of all 500 shots. Then we converted the spatial-pointing jitter in unit-of-length (Δ*x, y*_*uncor*_) to unit-of-angle (Δ*x*′, *y*′_*uncor*_) for the following reasons: (1) to make it easy to compare among different UED beamlines with different lengths; (2) to make it directly comparable with the electron beam divergence. This conversion is done *via* dividing Δ*x*, *y*_*uncor*_ by the distance from the gun to the detector (*L*_*G2D*_ = 4.19 m): Δ*x*′, *y*′_*uncor*_ ≈ Δ*x*, *y*_*uncor*_/*L*_*G*2*D*_. The main source of the spatial-pointing jitter comes from the shot-to-shot variation of the electron bunch when it exits the gun.

**Figure 5:**
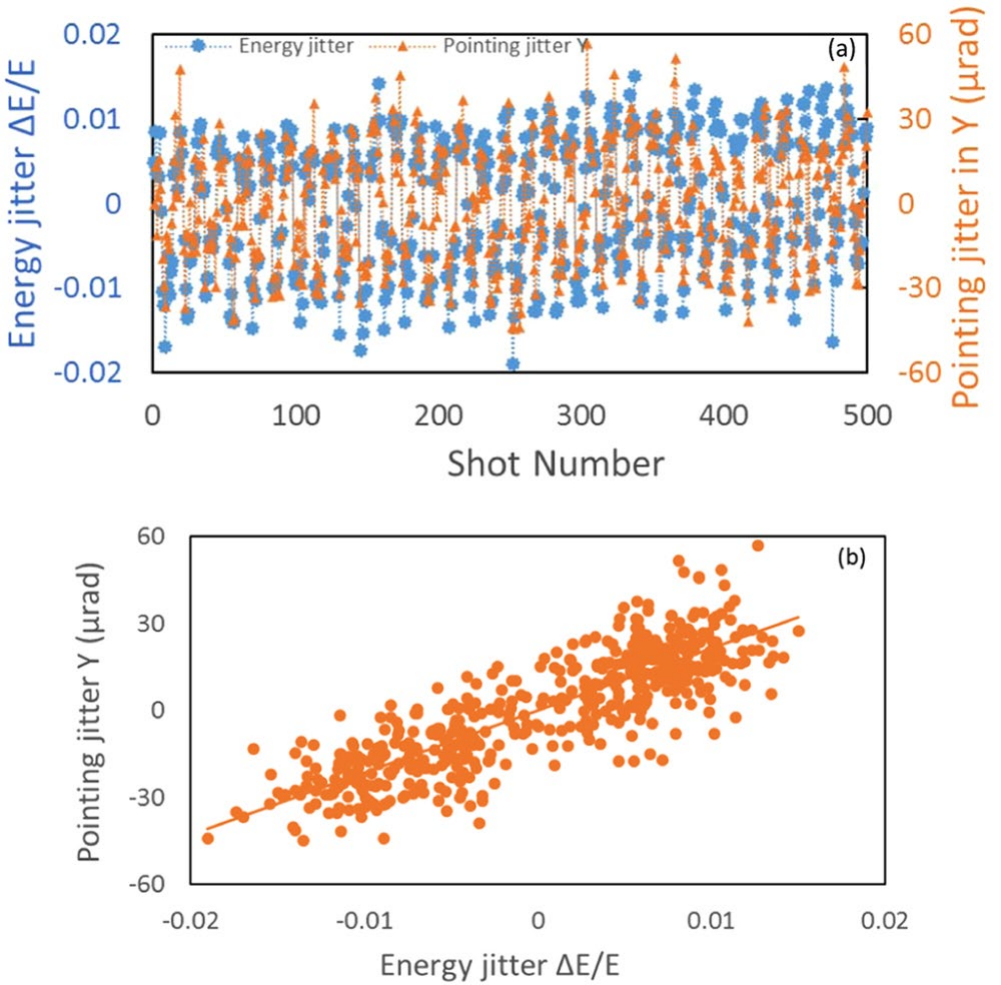
(**a**) (top) energy jitter (blue) and pointing jitter in y (orange) *vs* shot number are plotted as the primary y axis and secondary y axis respectively. (**b**) (bottom) correlation of the pointing jitter and energy jitter.

The correlation between the transverse motion of the diffraction image and the energy jitter shot-by-shot can be applied to measure the dispersion. Several sets of data with the large (about 2% peak-to-peak) shot-to-shot energy jitter were collected and analyzed. The dispersion measurement error is about 6% estimated as $\sqrt{{e}_{1}^{2}+{e}_{2}^{2}}$, where *e*_1_ = 0.02 is caused by different methods of data analysis and *e*_2_ = 0.06 is the statistical error. By averaging the ratio of the shot-to-shot spatial-pointing jitter (in unit of length) with respect to the energy jitter, we calculate the dispersion using the equation $\Delta y=\Delta y^{\prime} \cdot {L}_{G2D}={\eta }_{y}\cdot \frac{\Delta E}{E}$. The analysis of the data shown in Fig. 5(a) gives *η*_*y*_ ≈ 0.0098 m with a standard deviation of 6%. The same data set of Fig. 5(a) is plotted in the format of spatial-pointing jitter (in unit-of-angle) *vs* energy jitter as Fig. 5(b). From the x-y correlation analysis of Fig. 5(b) and multiplying this correlation (slope) by *L*_*G2D*_, we extract *η*_*y*_ ≈ 0.010 m. The difference of *η*_*y*_ is caused by the systematic error of applying different data analysis methods to the same data set. Similarly, we obtain *η*_*x*_ ≈ 0.004 m (10% error).

**Figure 6:**
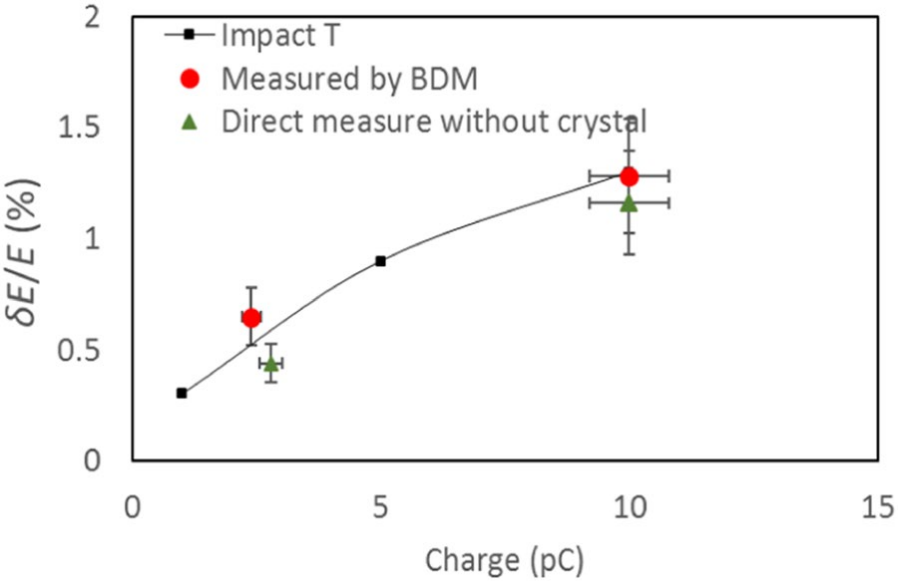
The electron beam energy spread measured by the BDM (red circles) and by direct beam size measurement (green triangles). They agree well with Impact-T simulations (black squares). The horizontal error comes mainly from the laser power fluctuation. The vertical error is described in the text.

It is a reasonable assumption based on the earlier experimental result^10^, the beam emittance *ε*_*x*_ ≈ *ε*_*y*_ and the beta function *β*_*x*_ ≈ *β*_*y*_. The beam size is defined as ${\sigma }_{x,y}=\sqrt{{\beta }_{x,y}{\varepsilon }_{x,y}+{({\eta }_{x,y}\frac{\delta {\rm E}}{{\rm E}})}^{2}}$ ^25^. The Debye-Waller factor doesn’t affect the Bragg peak width and position, only varying the peak intensities^26,27^. The differences of Bragg peak widths *σ*_*x*_ and *σ*_*x*_ are caused by different dispersions *η*_*x*_ and *η*_*y*_ and the beam energy spread *δΕ*/*Ε*. Therefore, we can measure the beam energy spread *via* $\frac{\delta {\rm E}}{{\rm E}}=\sqrt{\frac{{\sigma }_{y}^{2}-{\sigma }_{x}^{2}}{{\eta }_{y}^{2}-{\eta }_{x}^{2}}}$ using the dispersion measured by the BDM. We compare the measurement based on the Bragg peak widths with the direct beam size measurement without the beam diffraction *via* the sample. The results are consistent and agree reasonably well with Impact-T simulations, as shown in Fig. 6. So, using BDM we can measure the shot-to-shot energy fluctuation $\frac{\Delta {\rm E}}{{\rm E}}$, dispersion *η*_*x,y*_, the spatial-pointing jitter and the beam energy spread $\frac{\delta {\rm E}}{{\rm E}}$.

**Figure 7:**
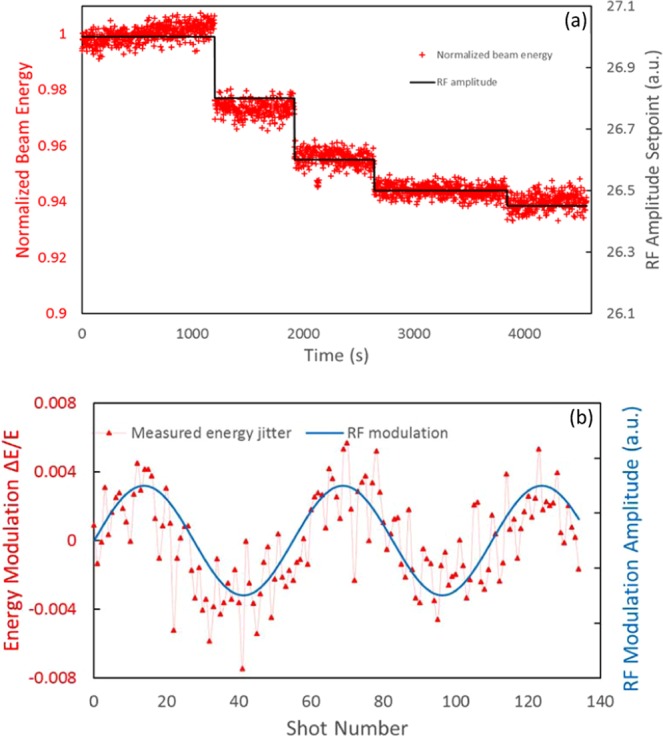
(**a**) (top) Normalized electron beam energy (red) and high voltage RF amplitude setpoint (black) are plotted as the preliminary y axis and secondary y axis respectively. (**b**) (bottom) Normalized electron beam energy (red) and LLRF modulator amplitude (blue) are plotted as the preliminary y axis and secondary y axis respectively.

Furthermore, we have applied BDM to calibrate the electron beam energy in real time by varying the RF voltage, the results are shown as Fig. 7(a).

We also applied a sine-wave modulation to the low-level RF (LLRF) input of the high-voltage RF amplifier and measured the beam energy variation *via* the BDM. The result is shown in Fig. 7(b). Modulating the amplitude of the LLRF drive signal varies only the RF amplitude and the phase seen by the beam is not changed. So, the measurement can be automated because there is no need for the phase correction during the measurement.

## Discussion

In conclusion, the novel BDM provides *in-situ* calibration of the electron beam such as shot-to-shot energy fluctuation and spatial-pointing jitter. The dispersion can be measured with a better precision when there is a large shot-to-shot energy oscillation (see e.g. Fig. 5). However, if the beam energy fluctuation is small, we can deliberately introduce a RF modulation with the desired amplitude (as demonstrated in Fig. 7(b)). This real time dispersion measurement enables online minimization of the dispersion at the detector therefore less dispersive jitter in the drift mode *via* optimizing the orbit corrector settings. The electron beam stability can be measured by the BDM without perturbing the UED experiment, unlike the conventional method based on the beam deflection by a dipole magnet. With the unique combination of nondestructiveness and capability of simultaneous measurement of the electron beam energy, energy spread and position, the BDM enables online optimization of the beam parameters. This is especially important for future high-charge single-shot UED and UEM developments.

The BDM can help in calibrating the shot-to-shot energy fluctuation with 10^−4^ precision. This real-time energy measurement enables the filtering process to remove off-energy shots improving the resolution *via* the offline accumulation of the filtered images. In the UED case, the filtering process can be replaced by rescaling the diffraction pattern using the real time measured beam energy. Also, the BDM can be a powerful tool to diagnose the RF problem. The electron beam with a relative energy spread ≤10^−4^ is required by many UEM and UED applications. E.g., to achieve the nanometer spatial resolution in the future UEM development, the energy spread of the electron beam must be kept to the 10^−4^–10^−5^ level for the purpose of minimizing the contribution from the dominating terms of chromatic aberrations^12^. The existing way to achieve the 10^−4^ energy spread of the electron beam is to operate the experiment in the low-charge (tens fC) mode with the minimized space charge effect, the compensated correlated energy spread *via* an additional RF harmonic cavity and optimized gun parameters^15,28,29^. There are a lot of efforts in the past a few years spent in the development of low energy-spread MeV RF gun. A promising superconducting RF gun providing the 10^−4^ energy spread and a pico-coulomb charge is in the process of being built^30^.

We plan to install a quadrupole triplet downstream the mirror which reflects the diffraction pattern vertically to the detector. A standard quadrupole scan, with the mirror set temporarily removed and an aperture installed to allow only the primary beam to go through, can provide the missing information of the beam emittance to make the online diagnostic package complete^31^.

It is worth noting that the BDM can be readily applied to UEM experiments. The only difference is that, for UEM experiments, the setting of the lenses has to be changed to image the diffraction pattern on the detector in order to apply the BDM. This is routinely done in conventional TEM to record images of both the real and the reciprocal spaces.

## Methods

Our method to monitor the shot-to-shot electron beam energy and spatial-pointing jitter in real time is based on a novel nondestructive Bragg-diffraction method. The information of the electron beam energy, energy fluctuation and spatial-pointing jitter is intrinsically encoded to the shot-to-shot diffraction image. Without perturbing the ongoing experiment, one can simultaneously measure the shot-to-shot energy fluctuation and spatial-pointing jitter of the electron beam in real-time *via* eigen-decomposing the variation of the diffraction pattern to two decoupled modes, the distance between the Bragg peaks as well as its variation (radial mode) and the overall lateral shift of the whole pattern (drift mode). The uncertainty coming from the fitted error of the center positions of those Bragg peaks determines the ultimate measured energy resolution, which is 2 10^−4^ in our case. The dispersion at the detector obtained *via* analyzing the correlation between the shot-to-shot spatial-pointing jitter and energy fluctuation can be applied together with the Bragg peak width to extract the electron beam energy spread information.
